# Prevalence and correlates of psychological distress symptoms among patients with substance use disorders in drug rehabilitation centers in urban Nepal: a cross-sectional study

**DOI:** 10.1186/s12888-016-1003-6

**Published:** 2016-09-08

**Authors:** Bishal Gyawali, Bishnu P. Choulagai, Damaru Prasad Paneru, Meraj Ahmad, Anja Leppin, Per Kallestrup

**Affiliations:** 1 Center for Global Health, Department of Public Health, Aarhus University, Bartholins Allé 2, Building 1261, 2:15, DK-8000 Aarhus C, Denmark; 2 Nepal Development Society (NEDS), Bharatpur, Nepal; 3 Department of Community Medicine and Public Health, Institute of Medicine, Tribhuvan University, Maharajgunj Kathmandu, Nepal; 4 Department of Public Health, School of Health and Allied Sciences, Pokhara University, Pokhara, Nepal; 5 Department of Community Medicine, Manipal College of Medical Sciences, Pokhara, Nepal; 6 Unit for Health Promotion Research, University of Southern Denmark, Niels Bohrs Vej 9, DK-6700 Esbjerg, Denmark

**Keywords:** Substance use disorders, Distress, Kessler-6-item scale, Rehabilitation, Nepal

## Abstract

**Background:**

The burden of substance misuse in developing countries is large and increasing, with negative consequences for physical and psychological health. Substance use disorders and psychological distress commonly co-exist, however few studies have examined this relationship in developing countries, including Nepal. Our aim was to investigate the prevalence of psychological distress symptoms and associated factors among patients with substance use disorders attending drug rehabilitation centers in Nepal.

**Methods:**

We conducted a cross-sectional study including 180 patients attending drug rehabilitation centers in the Kathmandu Valley region of Nepal. We used the 6-item Kessler scale (K6) to measure symptoms of psychological distress, and data on socio-demographics, behavioral and psychosocial factors. Multivariable analyses were used to identify factors associated with distress.

**Results:**

The prevalence of high psychological distress symptoms among patients with substance use disorder was 51.1 %. The mean score found on the K6 was 12.22 (SD = 5.87). Outcomes of multivariable analyses demonstrated various factors associated with symptoms of psychological distress, including age (β = −0.122, 95 % CI = −0.218; −0.026), education (β =2.694, 95 % CI = 0.274; 5.115), severity of drug abuse (Drug Abuse Screening Test-10-DAST10)(β = 0.262, 95 % CI = 0.022;0.502), and family functioning (Adaptability, Partnership, Growth, Affection and Resolve-APGAR) (β = −0.525, 95 % CI = −0.787; −0.264).

**Conclusions:**

High psychological distress symptoms are common in patients with substance use disorder in Nepal. Demographics (age, education), behavioral (drug abuse severity), and psychosocial factors (family functionality) were associated with psychological distress symptoms. If confirmed by future longitudinal studies such characteristics may assist in identifying groups at risk for co-morbid psychological distress symptoms among patients with substance use disorders. Future treatment approaches for substance use disorders should address co-existing mental illness in Nepal.

## Background

Substance use disorders (SUDs), according to DSM-5, refer to a problematic pattern of use of alcohol or another substance causing clinically significant impairment in daily life or noticeable distress. These disorders are prevalent worldwide and may lead to a wide range of physical, psychological and emotional health problems. SUDs are often associated with considerable psychiatric disorders, including depression and anxiety [1]. Various epidemiological [2, 3] as well as clinic based [4, 5] studies from Western countries have reported a high prevalence of comorbidity of SUDs and psychiatric disorders. Mental disorders and SUDs were one of the leading causes of disease burden in 2010 accounting for 7.4 % of global Disability-Adjusted Life Years (DALYs) and 22.9 % of global Years Lived with Disability (YLDs) [6]. This is a particular challenge for developing countries where the burden of mental health and SUDs is predicted to increase [6] but which-due to resource restrictions and limited prioritization in health care planning and workforce development-often face problems in fully addressing these problems. This is also the case for Nepal. Data about prevalence of mental health problems in population groups with SUDs are limited. However, a recent study of 188 consecutively admitted alcohol use disorder (AUD) patients in treatment facilities in Kathmandu found the lifetime and 12-month prevalence rates of major depression to be 45 and 36 % respectively [7]. In another smaller-scale cross-sectional study of alcohol-dependent patients in Eastern Nepal, 38 out of 60 patients (63.3 %) had one or more psychiatric disorders [8].

This substantial level of psychiatric comorbidity raises concern since depression and anxiety may not only influence help-seeking behavior, such as obtaining diagnosis and treatment for SUDs [9] as well as adherence to treatment [10]; they may decrease quality of life [11], increase risk of relapse [12], of social isolation [13] and risk of early mortality [14]. Risk factors for depression and anxiety among SUD patients in Western countries have been reported to be female gender [15], younger age, low family income and being single [16].

In Nepal, mental health risk factors in groups with SUDs have so far rarely been investigated. However, Neupane et al. in a study of 188 Nepalese patients being treated for AUDs reported that a main factor to be associated with major depression was not being married [7]. A similar finding was also reported by an earlier small-scale study by Pradhan et al. [17]. Further, coming from an urban as compared to a rural area and living in a nuclear family compared to a joint or extended family were positively associated with risk of depression while age, gender, education and income were unrelated [7]. However, there is a general paucity of information regarding potential behavioral and psychosocial correlates of psychological distress, and particularly regarding the factors, which potentially mediate associations between socio-demographic characteristics and mental health for instance, stress experience, or familial functionality. Thus, while family type and marital status have been reported to be relevant [7, 17], quality of family relationships might be just as or even more important. A study conducted in India, for instance, reported associations between poor relationships with family, poor physical health, experiences of violence and sexual abuse with depression and anxiety [18]. Another factor, which might be relevant in the socio-cultural context of Nepal, is the stigma involved in substance use/abuse. Neupane et al. in a study on treatment seeking for alcohol problems in Nepal found considerable differences in searching for treatment depending on whether alcohol consumption was considered a social taboo or not [19]. Similarly, it might be expected that patients who see themselves as socially stigmatized by being treated for substance abuse in a clinic might be more likely to experience psychological distress.

Despite the publication of some studies highlighting the high levels of psychiatric disorders in patients with SUDs, there continues to be a dearth of evidence examining the associated factors of psychological distress in SUDs, especially in the Nepalese socio-cultural context. A greater understanding of the specific factors associated with psychological distress among patients with SUDs is however necessary to develop appropriate and effective interventions. The aim of this study is to investigate the prevalence of psychological distress symptoms and associated factors among patients with substance use disorders attending drug rehabilitation centers in Nepal.

## Methods

### Study design, population and setting

This was a cross sectional study using convenience sampling technique. Data were collected between March and July 2014 in seven drug and alcohol rehabilitation centers in Kathmandu Valley of Nepal. These centers provided a therapeutic environment for detoxification of patients with SUDs. The Kathmandu Valley has a population of approximately 2.5 million, the majority of who are Hindus and Buddhists, while Muslims and Christians are religious minorities [20] Ethnic and caste groups comprise Dalits, disadvantaged and relatively advantaged Janajatis, disadvantaged non-Dalits, religious minorities, and upper caste groups [21]. The literacy rate in Kathmandu is 86.3 %, and Nepali is the most widely spoken language [20]. Mental health care is provided to some extent at the outpatient mental health facilities, although private clinics and clinics funded by Non-Government Organizations (NGOs) also exist [22]. Only few NGOs/private organizations in urban areas run rehabilitation services that include therapeutic detoxification and 12-step programs. At the time of the study, each rehabilitation center was providing care for between 30 and 90 patients.

### Data collection tools

Data on demographics, socio-economic conditions, behavioral (alcohol abuse severity and drug abuse severity), psychosocial factors (stressful life events, perceived stigma, and family functionality) and psychological distress symptoms were collected using a self-administered questionnaire. Prior to questionnaire development, an extensive literature review was conducted to identify associated factors for psychological distress.

We used a standardized questionnaire to collect socio-demographic information, including age, gender, ethnicity, marital status, education, occupation, monthly income and living situation. Age and monthly income were assessed as continuous variables. Ethnicity was grouped into three categories (Upper caste, Janajati and Others). Marital status was dichotomized into married and unmarried. Education was grouped into three categories (primary, secondary and university). Occupation was dichotomized into employed and unemployed. Living situation was dichotomized into living alone and with family. Stressful life events were defined as whether or not any serious personal or emotional problem had occurred during the past 12 months (no/yes).

We used the Kessler-6 (K6) Psychological Distress Scale to measure distress symptoms, a standardized and validated screening tool for non-specific psychological distress, including depression and anxiety [23]. The K6 offers the advantage of being a broader screening tool compared to some of the other locally validated mental health screens such as the General Health Questionnaire-12 (GHQ-12), and Patients Health Questionnaire-9 (PHQ-9), because it is not specific to a single disorder and has been validated to screen for common disorders in developing country settings [24]. The scale is available in a Nepali version [25] and has been used previously in Nepal [26], as well as in a variety of cultural settings in different parts of the world [27, 28]. The K6 uses a Likert scale to establish how often an individual has experienced psychological distress over the preceding 30 days. Scores range from 0 to 24 with higher score indicating better outcomes. A K6 score of greater than 12 has been defined as indicating high psychological distress [29]. Psychological distress assessment was performed on the seventh day after patients’ admission to the rehabilitation center.

We assessed participants’ risk of alcohol abuse using the Alcohol Use Disorder Identification Test (AUDIT), a 10-item screening measure designed to identify drinkers at risk for alcohol abuse or dependence [30]. Scores ranged from 0 to 40: 0–7 indicates low risk; 8–15 indicates moderate risk or hazardous drinking; 16–19 indicates high-risk or harmful drinking; and a score of 20 or above indicates possible dependence [30].

Drug use was measured using the Drug Abuse Screening Test-10 (DAST-10), which quantitatively assesses the severity of drug-related problems. A single summary score reflects the number of drug abuse items endorsed. A score ranging from 1 to 2 indicates low risk, 3–5 indicates moderate severity level, 6–8 indicates a substantial level and a score ranging from 9 to 10 indicates a severe level of problems [31].

We used the Perceived Stigma of Substance Abuse Scale (PSAS) which is an eight item self rated scale to look at stigma experienced by the substance users due to their substance use [32]. All items are marked on a Likert-type scale with four options (‘totally disagree’ to ‘totally agree’). Six of the eight items are reverse scored. The scale ranges from 8 to 12, higher scores indicating presence of perceived stigma. Luoma et al. developed and validated the scale which is easy to administer and has shown a good face and construct validity and an acceptable internal consistency [32].

Perceived family functionality was measured by the Adaptability, Partnership, Growth, Affection and Resolve (APGAR) Scale, which comprises questions about key components of family function [33]. A score of 7 to 10 suggests a highly functional family, a score of 4 to 6 suggest a moderately dysfunctional family and a score of 0 to 3 suggests a severely dysfunctional family.

### Procedure

Three specially trained research assistants with a health professional background carried out the administration of the questionnaires in training rooms available in each center. The research assistants introduced themselves at first and then briefly explained the purpose of the study. Subsequently, an informed consent form was distributed to all eligible participants and later collected. Eligible participants were patients undergoing residential treatment for SUDs, including alcohol abuse and drug abuse who were 16 years or above, and able to read and write in Nepali. Those who were reluctant to participate in the study and who had cognitive impairments were excluded from the study. Presence of cognitive impairment was assessed by asking patients whether they had ever been diagnosed by a physician as having memory problems. In case of any literacy difficulty, the consent form was read aloud. The consent form informed participants about the purpose of the study and advised that participation was voluntary. Participants were assured verbally and on paper that all information provided would be kept strictly confidential and not used except for the study purpose. All participants who agreed to participate were provided an informed consent form to sign. After obtaining a written informed consent, the research assistants distributed the self-administered questionnaires and offered necessary instructions for the participants to fill it out anonymously. The study protocol received ethical approval from the Manipal College of Medical Sciences, Pokhara, Nepal.

### Statistical analysis

**Fig. 1:**
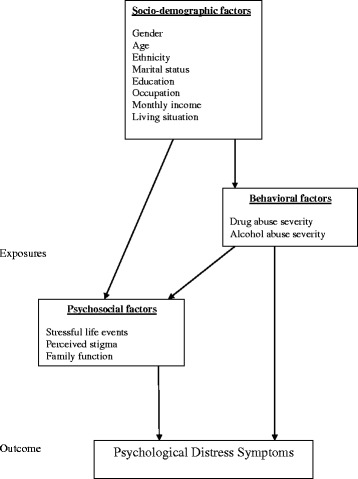
An analytical conceptual framework of associated factors for psychological distress symptoms

We conducted descriptive analyses to identify characteristics of the study sample. We analyzed the K6 data as a continuous outcome. Analyses of associated factors were conducted in two stages: i) the association of each variable with distress was assessed through univariate linear regression models; ii) multivariate analyses were carried out in the second stage among variables that showed an association at *p* < 0.05 in the first stage. To guide these multivariable analyses, we used an analytical framework based on hierarchical relationships of factors associated with Psychological Distress Symptoms Fig. 1.

This approach enabled us to take account of hierarchical relationships between predictors, and balance reliance on statistically significant associations [34]. Furthermore, we arranged associated factors into three levels: the top level consisted of socio-demographic factors, which we considered to act directly, or indirectly through intermediate factors to cause distress; the next level included behavioral factors; the lowest level included psycho-social factors as the most proximal predictors. Correlation analyses and t-tests were used to identify significant factors associated with psychological distress. Reliability was assessed with Cronbach’s alpha [35]. All statistical analyses were conducted using Statistical Package for Social Science 22.0 (SPSS 22.0) software [36].

According to the framework, groups of variables were entered in hierarchical order into a multivariable modeling procedure. A similar approach has been used previously to assess predictors of common mental disorders [37, 38]. The assumptions of linear regression (outliers, normality of distribution and linearity) were checked (using box plot, histogram, q-q plot and scatter plot). Tolerances for the parameter estimates were examined to assure that multicollinearity was not a problem.

## Results

All of the 180 participants completed and returned the questionnaire. Socio-demographic characteristics of participants are summarized in Table 1. The study population was mostly males (85.6 %) and the mean age of the participants was 29.08 years (SD ± 9.98). More than half of participants (51.7 %) belonged to Upper Caste Groups and 54 % were unmarried. More than 52 % of the participants were unemployed. Nearly 88 % were living with family. The average monthly income of participant’s household was Rupees 23,030.60 (SD ± 8628.54) or 230.31 US Dollars (1 Nepali Rupees = 0.01 US Dollar, July 2014). The mean DAST score was 5.42 points (SD = 3.918). The internal consistency of the DAST scale was found to be α =0.86. The mean AUDIT score was 17.03 points (SD = 12.078) and Cronbach’s alpha for the scale was 0.85. The mean on the perceived stigma scale was 24.32 points (SD = 5.095). Internal consistency of the stigma scale was α = 0.72. The mean APGAR score was 5.69 points (SD = 2.907). The internal consistency of the APGAR scale was α = 0.84. The K6 score distribution was approximating a normal distribution with a mean score of 12.22 and standard deviation (SD) of 5.87. The mean K6 score for male and female participants were 11.84 ± 5.91 and 14.42 ± 5.18 respectively. The internal consistency of the K6 was Cronbach’s alpha = 0.89. The overall prevalence of high psychological distress symptoms among participants was 51.1 % (*n* = 92).

**Table 1:** Sample characteristics

Characteristics	M or n	% or SD
Age	29.08	9.981
Gender		
Male	154	85.6
Female	26	14.4
Ethnicity		
Upper caste	93	51.7
Janajati	82	45.6
Others	5	2.8
Marital status		
Married	83	46.1
Unmarried	97	53.9
Education		
Primary	20	11.1
Secondary	114	63.3
University	46	25.6
Occupation		
Employed	85	47.2
Unemployed	95	52.8
Living situation		
Alone	22	12.2
With family	158	87.8
Stressful life events		
Yes	116	64.4
No	64	35.6
Monthly income (Rupees)	23,030.60	8628.54
Drug abuse severity (DAST-10)	5.42	3.918
Alcohol abuse severity (AUDIT)	17.03	12.078
Perceived stigma	24.32	5.095
Family functioning (APGAR)	5.69	2.907

Note: *M* mean, *n* group size, *SD* standard deviation, *DAST-10* drug abuse screening test-10, *AUDIT* alcohol use disorder identification test; *APGAR* adaptability, partnership, growth, affection and resolve

Univariate correlational analyses and t-tests were performed initially between individual K-6 scale scores and three types of variables: socio-demographic variables (age, gender, ethnicity, marital status, education, occupation, living situation, and monthly income), behavioral variables (severity of substance abuse: scores on DAST and AUDIT) and psychosocial variables (stressful life events, perceived stigma, and scores on APGAR) (Table 2). We found significant correlations and t-test differences between the K6 scores and age, gender, marital status, education, score on DAST, perceived stigma, and APGAR.

**Table 2:** Univariate analysis of K6 scores in relation to socio-demographic characteristics, behavioral characteristics, and psychosocial characteristics

*N* = 180	M or n	% or SD	t	r	*P*-value
Socio-demographic characteristics					
Age (years)	29.08	9.981		−0.3	*p* < 0.001
Female	26	(14.4 %)	1.99		0.048
Upper Caste	93	(51.7 %)	1.127		0.256
Unmarried	97	(53.9 %)	2.031		0.044
Primary education	20	(11.1 %)	2.226		0.027
Unemployed	95	(52.8 %)	1.776		0.078
Monthly income	23,030.60	8628.54		- 0.39	0.700
Living alone	22	(12.2 %)	1.247		0.313
Behavioral characteristics					
Alcohol abuse severity	17.03	12.078		0.127	0.136
Drug abuse severity	5.42	3.918		0.391	*p* < 0.001
Psychosocial characteristics					
Stressful life events	116	(64.4 %)	0.703		0.483
Perceived stigma	24.32	5.095		0.339	*p* < 0.001
Family functionality	5.69	2.907		0.315	*p* < 0.001

Note: *t* observed t value, *r* correlation, *P* significance level

We, then, performed hierarchical multiple regression analyses with those socio-demographic variables, behavioral variables, and psychosocial variables that had been significant in the univariate analyses as predictors and the K-6 score as the dependent variable (Table 3). The socio-demographic variables age, gender, marital status, and education were entered into the first block and accounted for 13.4 % of the variance in K6 scores [F (4,175) = 6.788]. The second block included the DAST score and explained an additional 4.1 % of the variance [F (5,174) =7.383]. Block three added perceived stigma and the APGAR score and explained an additional 8.7 % of the variance [F (7,172) =8.728].

**Table 3:** A hierarchical multiple regression analysis of determinants of K6-scores: contributions of each variable block to changes in R^2^

Determinants		R^2^	Δ R^2^	F	df	ΔF	Sig ΔF
Block 1	Socio-demographics	0.134	0.134	6.788	4,175	6.788	*p* < 0.001
Block 2	Behavioral	0.175	0.041	8.587	5,174	7.383	0.004
Block 3	Psychosocial	0.262	0.087	10.148	7,172	8.728	*p* < 0.001

Note: R^2^, F and df describe the overall regression equation after each block has been entered into the model. ΔR^2^, ΔF, and significance ΔF describe the contributions of each individual block

Summary statistics for the complete model are presented in Table 4. The final regression model accounted for a significant 26.2 % of the explained variance in K6 score [F (7,172) =10.14].

**Table 4:** Multivariate analysis of K6 scores

Determinants	β (95 % CI)	SE	*P*-value
Age	−0.122 (−0.218; −0.026)	0.049	0.013
Female	1.206 (−0.983; 3.394)	1.109	0.278
Unmarried	−0.610 (−2.421; 1.202)	0.918	0.507
Primary education	2.694 (0.274; 5.115)	1.226	0.029
Drug abuse severity	0.262 (0.022; 0.502)	0.122	0.032
Family functionality	−0.525 (−0.787; −0.264)	0.132	*p* < 0.001

Note: *β* regression coefficient, *CI* confidence interval, *SE* standard error

Age (β = −0.12; 95 % CI −0.218, −0.026; *p* = 0.013) and education (β = 2.694; 95 % CI = 0.274, 5.115, *p* = 0.029) were the only socio-demographic variables that were significant predictors in this model. In addition, scores on the DAST (β = 0.262; 95 % CI = 0.022, 0.502; *p* = 0.032) and the APGAR (β = −0.525; 95 % CI = −0.787, −0.264; *p* < 0.001) were significantly related to the K6 score in the complete model.

## Discussion

The present results show that 51.1 % of patients being treated for SUDs in Kathmandu Valley treatment centers experienced high psychological distress symptoms. As expected this rate is considerably above those found in community settings for the general population of Nepal, where studies have reported psychological distress rates from 9.8 % [39] to 33.7 % [40]. Two other studies have specifically reported distress data for patients treated for SUDs in Nepal, one reporting lower, the other considerably higher distress prevalence. In the study by Neupane et al. only 51 (30.2 %) out of 169 patients with AUD had recently experienced depressive symptoms as measured by the Hopkins Symptoms Check List-25 [37] and 36 % had experienced at least one major depressive episode assessed by the WHO’s Composite International Diagnostic Interview in the preceding 12 months [7]. A main factor explaining the discrepancy between prevalence rates in the two studies may lie in the different time window for distress prevalence estimation. While the present study assessed distress at the end of the first week of treatment asking for experience in the preceding four weeks, the study by Neupane et al. assessed symptoms during the preceding two weeks excluding the week post-admission, since the later is specifically stressful, difficult and often painful period. Including distress ratings from this period may, due to their transitory nature, lead to overestimation of longer-term distress experience. Further, the present study included slightly higher proportions of younger respondents who are known to report higher distress rates [37, 41]. Another study from Nepal by Pradhan et al., including 53 patients treated from AUD, found as many as 94.3 % of patients suffering from depressive symptoms as measured by the Hamilton Depression Rating Scale [17]. This extremely high rate is explained by the fact that the study included only patients who had been admitted to the psychiatric ward of the hospital with the diagnosis of mental or behavioral disorder due to alcohol use.

Comparisons with prevalence data from other countries also show a wide range of distress rates. Comparable findings to those of the present study where reported by a Jamaican study of 56 patients treated for SUDs [42], where 42.8 % reported severe or very severe depression symptoms on the Kessler-10- scale and a study from Hunan Province, China, where 43 % of heroin-dependent patients in a treatment center showed depressive symptoms and 53 % anxiety symptoms as assessed by the Zung’s Self-Rating Depression (SDS)/Self-Rating Anxiety Scales (SAS) [43].

In contrast, much higher prevalence rates were reported by a Norwegian study, which found that 83 % of 185 in patients treated for alcohol and other drug abuse, scored above the cut-off for the Hopkins Symptoms Checklist-10 [44]. Distress prevalence was assessed for the week prior to treatment admission, a period, where psychological problems might culminate. Armstrong et al. in a study on 420 drug injecting male users participating in needle-and syringe programs in Delhi, India also found extremely high rates of 84 % of participants with depressive and 71 % with anxiety symptoms measured by the PHQ-9 [18]. In this case the study population was considerably more socially disadvantaged than the present study, with high proportions being illiterate, homeless and living with very small income/being dependent on scavenging.

For the present study it might be argued that given the timing and reference period for distress assessment, the prevalence rate particularly reflects the intensely stressful phases before entering treatment and early days of treatment, this overestimating distress levels in terms of a longer-term and more stable experience in need of psychiatric/psychological treatment. Despite this caveat, proportions of patients suffering from psychological comorbidity are sizeable. And while it remains unclear to which extent development of SUDs and/or being in treatment for SUDs causes distress or prior distress and depression contribute to self-medication via substance use, the findings indicate a need for action, since these conditions, if left untreated are likely to increase the risk of poor health outcomes, of relapse after treatment, workplace productivity loss and even premature mortality [45, 46].

Our study also investigated a set of socio-demographic, behavioral, and psychosocial factors that may be important to consider in understanding the development of psychological distress symptoms among this population. In particular, we found that family functioning as measured by the APGAR-Scale was significantly negatively associated with the K6 distress score. Prior studies conducted in Western and Eastern countries have also reported that family dysfunction is strongly associated with mental disorders [47, 48]. Few studies, however, have so far provided support for the association between family functioning and psychiatric comorbidity in Nepal [49] and India [50]. A study by Krishnakumar et al. [51] in India revealed that sources of distress in the family could be parental deaths, inter-parental conflicts, unfulfilled needs and wants, mental illness, parental substance addiction, parental divorce and disharmony. That the family history of substance abuse might be a relevant factor, was also shown in a study by Neupane et al. with Nepalese patients being treated for AUD and where reported that parental problem drinking was related to major depression in social groups where alcohol consumption was considered taboo [7]. Deficits in family functioning may lead to experience of distress and subsequent increase in substance use. Moreover, Hosseinbor et al. found that individuals who do not have an intimate and supportive relationship within their family are more likely to be attracted by and inclined towards friends and groups of their own age and become more susceptible to using drugs in the social context of these peer groups, which again later may cause psychological problems [52].

Vice versa SUDs in a family member will very likely affect the family system and family relations in a negative way so that conflicts arise and relations deteriorate over time. Shyangwa et al. in a study on the families of patients with opium dependence syndrome in New Delhi showed that 75 % of patients’ spouses perceived a severe burden due to their husband’s opioid dependence [53].

This study further demonstrated that having only primary education is associated with more psychological distress symptoms. This finding is consistent with the previous studies [54, 55]. People with less education may feel socially trapped and helpless and might experience more adverse situations which could directly contribute to the emergence of psychological distress [56]. Further, patients with lower educational attainment may have lesser knowledge and poorer coping skills to deal with their substance abuse problem and thereby increase psychological distress and poor quality of life.

Also, age was a significant and negative correlate of psychological distress symptoms suggesting that as age increases, the level of psychological distress decreases. This is supported by another study conducted in Nepal, investigating recent depressive symptoms in AUD patients [37]. In general, young age is an unstable period in life when one may be less able to deal with daily stress while coping with developmental tasks, particularly when during puberty major physical an psychological changes are experienced [37, 46]. Evidence also shows that family disputes and domestic violence in Nepal place youngsters at greater risk of distress [57]. Furthermore, age also affect distress via severity of drug use since drug use was highest in the younger group and severity of drug use was again associated with distress. The association between psychological distress and age underscores the need for detection, assessment and treatment of mental health problems at early ages among patients with SUDs.

Finally, this study found that drug use severity as measured by the DAST-10 was significantly positively associated with psychological distress symptoms as also reported elsewhere [44]. Higher drug consumption may lead to higher levels of compromised physical health, negative social consequences as well as self-esteem problems while higher levels of distress experience might also lead to higher drug usage levels. Further studies should use prospective data to identify pathways that connect family dysfunction, drug use, and psychological distress. Severity of alcohol use on the other hand was neither a univariate nor an independent correlate of distress. A possible reason for this absence of an association could be lack of variability in alcohol use, since non-use of alcohol was relatively infrequent in our study.

Gender and marital status, while being significant on the univariate level, no longer predicted psychological distress in the multivariate regression analysis. These factors very likely are associated with some of the other influences, particularly severity of drug consumption as well as family functioning. Once these more direct influence factors are entered into the equation, the more distal indicators loose relevance. Similarly, perceived stigma was significantly positively related to distress in the bivariate analysis, but there was no independent effect in the multivariable model. Here it is presumably the effect of family functionality, which makes a difference, since perception of stigma might be more likely where SUD patients experience conflictual relationships with their families.

Also, somewhat surprisingly, stressful events were unrelated to distress experience. However, it is important to consider that the study population was already highly strained group. When being admitted to a treatment center, many might have had a long past trajectory of drug abuse, social conflicts or economic problems with ensuing self worth problems behind them. Singular negative events might have relatively less to add to distress in such samples than in average groups of citizens. For such a highly burdened population group a more detailed assessment than the dichotomous question used in the present study might have generated different results. Thus, Liao et al. in a study with patients treated for heroin dependence in China used a detailed life event rating scale, explicitly listing different types of events, and found significant associations with anxiety and depression [43].

### Strengths and limitations

This is one of the few studies, which surveyed psychological distress symptoms among patients with SUD in Nepal. Data were collected using standardized instruments, which strengthened the reliability of the collected data. However, several limitations of this study should be considered. First, the analyses were conducted with cross-sectional data and thus, no causal statements could be made. Secondly, data in our study were based on self-report and no clinical assessments or biomarkers, for instance, to investigate the history of drug and alcohol use in the recent past were included. Third, the study was limited by a lack of validation of the mental health outcome tools within a Nepalese population. Fourth, as already pointed out above, the chosen time reference for distress assessment may have led to overrepresentation of transitorily high distress experience due to acute crisis, withdrawal symptoms etc. Such a strong influence of situational effects might also have limited the possibility to detect associations between patient characteristics or social influences and distress experience. A fifth limitation involves sampling bias as the clinics in the study were conveniently selected and restricted to the urban Kathmandu valley area in the first place, thus restricting possibilities to generalize our findings to all substance users in Nepal. Finally, we did not conduct gender-specific analyses because it would have involved too small sub-samples (especially the female sample).

## Conclusions

Psychological distress is a serious problem among patients with SUD in Nepal. Clinicians working with SUD patients should consider co-morbid psychiatric illness and therefore this group of population should routinely be screened for psychiatric co-morbidity. Younger age, lower levels of education, higher severity of drug abuse and lower family functionality may be important factors for psychological distress among SUDs in urban Nepal. If confirmed by future longitudinal studies such characteristics may assist in identifying groups at risk for co-morbid psychological distress symptoms among patients with SUDs. Future treatment approaches for substance abuse must address co-existing mental illness in Nepal. If the treatment of substance addiction is limited only to medical interventions, it is unlikely to attain full effect. Therefore, an integrated approach to treatment of SUDs, including supportive educational strategies and interventions to teach coping skills in preventing substance addiction and psychological distress may be warranted. However, more research needs to be conducted to substantiate these findings and to better inform intervention studies aimed at improving social, behavioral and psychosocial functioning of patients with SUD. Moreover, longitudinal studies examining the factors associated with developing psychological distress among patients with SUD will be helpful in disentangling causal relations and identifying at an early stage those who are at risk. Future research should also aim to index the K6-Scale in the Nepalese populations so that the tool and current data can be used to adequately estimate population prevalence of psychological distress.
